# Phenethyl isothiocyanate induces calcium mobilization and mitochondrial cell death pathway in cholangiocarcinoma KKU-M214 cells

**DOI:** 10.1186/1471-2407-13-571

**Published:** 2013-12-05

**Authors:** Ornanong Tusskorn, Laddawan Senggunprai, Auemduan Prawan, Upa Kukongviriyapan, Veerapol Kukongviriyapan

**Affiliations:** 1 Department of Pharmacology, Faculty of Medicine, Khon Kaen University, Mitraparb Road, Khon Kaen 40002, Thailand; 2 Liver Fluke and Cholangiocarcinoma Research Center, Khon Kaen University, Khon Kaen 40002, Thailand; 3 Department of Physiology, Faculty of Medicine, Khon Kaen University, Khon Kaen 40002, Thailand

**Keywords:** Phenethyl isothiocyanate, Anticancer, Cholangiocarcinoma, Mitochondrial transmembrane potential, GSH redox, Intracellular calcium

## Abstract

**Background:**

Phenethyl isothiocyanate (PEITC) is a cancer chemopreventive agent from cruciferous vegetables. Cholangiocarcinoma (CCA) is a chemo-resistant cancer with very poor prognosis. We evaluated the effects of PEITC on induction of apoptotic cell death in relation to cellular glutathione (GSH) and mitochondrial function of a CCA cell line, KKU-M214.

**Methods:**

Cytotoxic effects of PEITC on a CCA cell line, KKU-M214, and a reference cell line, Chang cells were evaluated. To delineate mechanisms of cell death, the following parameters were measured; GSH and superoxide levels as the oxidative status parameters, apoptosis related proteins levels using Western blotting. Cellular free calcium level and mitochondrial transmembrane potential were also measured.

**Results:**

PEITC induced apoptotic cell death of both KKU-M214 and Chang cells. After PEITC treatment, both cells showed decrease of Bcl-xl and increase of Bax levels. While KKU-M214 cells released AIF, Chang cells released cytochrome c, with subsequent activation of caspase 3 and 9, upon PEITC treatment. PEITC induced superoxide formation in both cells, although it seemed not play a role in cell death. PEITC caused GSH redox stress in different ways in two cell types, because *N*-acetylcysteine (NAC) prevented redox stress in Chang but not in KKU-M214 cells. The loss of mitochondrial transmembrane potential was induced by PEITC concurrent with GSH stress, but was not a primary cause of cell death. The rapid increase of free calcium level in cytosol was associated with cell death in both cell lines. These events were prevented by NAC in Chang cells, but not in KKU-M214 cells.

**Conclusion:**

PEITC induced cell death KKU-M214 cells and Chang cells via increase of cellular calcium mobilization and activation of mitochondrial cell death pathway. The effects of PEITC on the redox stress was mediated via different ways in CCA and Chang cells because NAC could prevent redox stress in Chang cells, but not in KKU-M214 cells. The multiple effects of PEITC may be useful for the development of novel chemotherapy for CCA.

## Background

Cholangiocarcinoma (CCA) is a malignancy originating from the bile ducts, usually adenocarcinomatous, and is the second common primary liver cancer [1]. CCA is a rare cancer worldwide, but the most common form of liver cancer in Mekong subregion countries, including northeastern Thailand, Cambodia, Vietnam and Laos [2]. In the Western countries, the incidence and the mortality rate of intrahepatic CCA have risen steeply and steadily over the last decades [3]. In spite of tremendous efforts to improve the treatment, CCA is still notoriously difficult in diagnosis and treatment [3]. Most of CCA patients are already in the advanced stage at diagnosis, and the radical surgery is not feasible. Chemotherapy and radiotherapy could not improve the survival of patients with unresectable CCA [3]. Despite of recent advances in chemotherapy for many cancers, management of CCA with chemotherapeutic drugs and biologic agents has so far been unsatisfied. The development of appropriate new chemotherapeutic drugs and new approaches for the treatment of chemo-resistant cancer like CCA should be of the high priority.

Isothiocyanates (ITCs) are the hydrolysis products of a group of naturally occurring thioglucoside and glucosinolate compounds found in cruciferous vegetables [4]. Among ITCs, phenethyl isothiocyanate (PEITC), sulforaphane and benzyl isothiocyanate are known to have potent biological activities. Recent epidemiological studies showed that intake of ITCs reduced the risk of certain cancers, such as pancreatic and lung cancers [5,6]. PEITC can suppress tumor cells growth, and induce apoptosis and cell cycle arrest [7]. Dietary intake of PEITC strongly inhibited tumorigenesis in various animal models such as a prostate cancer xenografted model [8], colon and lung tumors in transgenic mice models [9,10]. The effects of PEITC on tumor cells are multifaceted including induction of reactive oxygen species (ROS) formation, depolarization of mitochondrial transmembrane potential (∆*Ψ*_m_) [11,12], activation of c-Jun N-terminal kinase (JNK) and p38 kinase-mediated apoptotic pathway [7,13], and induction of hyper-expression of death receptor-5 mediated caspase 8 activation [14]. PEITC also can depress pro-survival signaling pathways of NF-κB and PI3K/Akt [15], as PEITC inhibits IKK, IκB and Akt phosphorylation leading to inhibit cell proliferation and apoptosis.

The selective cytotoxic effects of ITCs on various cell types have less frequently been reported. PEITC, benzyl ITC and sulforaphane showed the same ranges of IC_50_ values to MCF-7 breast cancer cells and MCF-12A, the non-cancer mammary epithelial cells, and also to HK-2 kidney cells [16]. The selective toxicity of anticancer agents on the major organs may be the primary concern in cancer chemotherapy. Although some functional distinction between cancer and normal cells such as oxidative status have been suggested [17,18], it is yet to be translated into an effective strategy to eliminate cancer cells while spare normal cells.

The effects of PEITC on multiple steps of cancer growth make this compound highly versatile and promising candidate in cancer chemoprevention and chemotherapy. However, the exact mechanisms of its chemopreventive effects are not fully understood. For the better understanding of the anticancer mechanisms of PEITC, we evaluated the effects of PEITC on a bile duct cancer cell line, KKU-M214 in relation to GSH redox stress and mitochondrial function. In this study, HeLa [Chang liver] cells were used as the reference because our previous study showed that oxidative stress caused the suppression of glutathione (GSH) redox in this cell line [19].

## Methods

### Cell cultures

Human CCA cell line, KKU-M214 established in our institute and human HeLa [Chang liver] cells were both kindly provided by Prof. Banchob Sripa, Department of Pathology, Faculty of Medicine, Khon Kaen University. Cells were grown in Ham’s F12 medium supplemented with 12.5 mM N-2-hydroxyethylpiperazine-N’-2-ethanesulfonic acid (HEPES; pH 7.3), 100 U/mL penicillin, 100 μg/mL streptomycin sulfate and 10% fetal calf serum and maintained under 5% CO_2_ in air at 37°C as described previously [20]. The cells were subcultured every 2–3 days before confluence of the cells using 0.25% trypsin–EDTA, and the medium was changed after an overnight incubation.

### Cytotoxicity assay

KKU-M214 and Chang liver cells were seeded onto 96-well culture plates at a density of 7,500 cells/well. After an overnight culture, the serum-free medium was replaced with the media containing PEITC, and the cells were cultured for various time intervals. The cytotoxicity was examined by the sulphorhodamine B (SRB) assay. In brief, cultured cells were washed once with phosphate-buffered saline (PBS), fixed with 100 μl of ice-cold trichloroacetic acid for 1 h, and stained with 50 μl of 0.4% SRB in 1% acetic acid for 30 min. The cells were then rinsed several times with 1% acetic acid, and protein-bound dye was dissolved with 200 μl of 10 mM Tris base solution. The absorbance was determined using a microplate reader with the filter wavelength of 540 nm.

To determine cells undergone apoptosis, cultured cells were stained with fluorescent dyes as previously described with modifications [20,21]. In brief, the medium was removed after PEITC treatment and the cells were stained with acridine orange and ethidium bromide (AO/EB) in PBS. The cells were examined using a Nikon Eclipse TS100 inverted microscope with the excitation and long-pass emission filters of 480 nm and 535 nm, respectively. The fluorescent images were taken at 2 predetermined areas in each well with triplicate wells per concentration using a Nikon Coolpix digital camera. The number of viable, apoptotic, and necrotic cells, which were stained with green fluorescence with intact nuclei, green fluorescence with the appearance of cell shrinkage, nuclear condensation and fragmentation, and bright orange fluorescence, respectively, were enumerated. The apoptotic cells were calculated as the percent apoptotic cells over a total number of cells in the same area.

### Measurement of ROS

Intracellular ROS generation was measured using a cell-permeable fluorescent probe, dihydroethidium (DHE). Briefly, 5 × 10^4^ cells were seeded in 96 black well plates and cultured overnight. Then, the medium was removed and the cells were washed with phosphate buffered saline (PBS). They were then treated with PEITC and 25 μM DHE with or without 2 mM *N*-acetyl-L-cysteine (NAC) or 0.5 mM 4-hydroxy-TEMPO (TEMPOL), in serum-free medium and kept in 5% CO_2_ atmosphere at 37°C for 90 min. The fluorescence intensity was read and quantified in a Gemini XPS fluorescent plate reader with the excitation and emission wavelength of 518 nm and 605 nm, respectively.

### Glutathione assay

Total glutathione was measured essentially according to Tietze’s method [22]. Glutathione disulfide (GSSG) was assayed by the method previously described [21] using 1-methyl-2 vinyl-pyridinium trifate (M2VP) as a glutathione scavenger. Cultured cells were trypsinized and washed three times with cold PBS. Cell suspensions were reacted with M2VP (1 mM) to determine GSSG. Aliquots of untreated cell suspensions were used for the assay of total GSH. Protein concentration was assayed using the Bradford’s dye binding method.

### Calcium mobilization assay

Intracellular calcium level was measured using an assay kit (FluoForte® Calcium Assay, Enzo Life Sciences, PA, USA). In brief, KKU-M214 and Chang cells were grown on 96 well plates at the density of 10,000 cells/well and treated with 3 and 10 μM PEITC with/without 2 mM of NAC for 1 h. Then, the media were removed and FluoForte® dye-loading solution was added according to the manufacturer’s instruction. The plate was incubated at 37°C for 30 min and the fluorescent staining was analyzed under a fluorescent microscope with the excitation and emission wavelengths of 485 nm and 535 nm, respectively. Images of the cultured cells were captured and the integrated optical density (IOD) of each image was analyzed by using the Image-Pro Plus (Media Cybernetics) software.

### Measurement of mitochondrial transmembrane potential

The dissipation of the mitochondrial electrochemical potential gradient is known as an early event leading to apoptosis. To measure the change in mitochondrial transmembrane potential (∆*Ψ*_m_), cells were seeded in 96 black well plates at the density of 10,000 cells/well and cultured overnight before treatment with various concentrations of PEITC for 3 and 24 h. The assay was performed according to the method described previously [23] using the cationic, lipophilic dye, 5,5′,6,′-tetrachloro-1,1′,3,3′tetraethylbenzimidazolyl carbocyanine iodide (JC-1) (Clayman Chemical) staining with some modifications. The cultured plate was centrifuged at 1,000 rpm for 5 min at room temperature and the cultured medium was removed, loaded with JC-1 dye for 20 min, washed by centrifugation, incubated in the assay buffer and ∆*Ψ*_m_ was analyzed under a fluorescent microscope with the excitation wavelength of 485 nm and emission wavelength of 535 nm. JC-1 forms J-aggregates in healthy mitochondrial matrix, which can be visualized as red fluorescence. In depolarized mitochondria, JC-1 effluxes to the cytoplasm and exists as monomers with green fluorescence. The shift of red to green fluorescence is an indicative of the depolarization of ∆*Ψ*_m_.

### Western blot analysis

Whole cell lysates were prepared as described previously [21]. PEITC-treated and control cells were washed with PBS, collected, and lysed with cell lysis buffer at 4°C with vigorous shaking. After centrifugation at 12,000 g for 30 min, the supernatant was collected and stored at -80°C until use. The protein samples (30 μg protein/sample) were electrophoretically separated on 10% SDS–polyacrylamide gel (SDS-PAGE). The proteins were transferred to polyvinylidene difluoride (PVDF) membranes by semi-dry blotting at 10 V for 40 min. The PVDF membranes were blocked for 2 h at 4°C with 5% (w/v) skimmed milk powder in PBS containing 0.1% Tween-20. The PVDF membrane was incubated overnight at 4°C with primary antibodies including rabbit polyclonal IgG against cytochrome c (sc-13560, Santa Cruz BioTechnology, 1:1000 dilution), mouse monoclonal IgG against Bcl-xl (sc-8392, 1:500 dilution), rabbit polyclonal IgG against Bax (sc-493,1:1000 dilution), rabbit polyclonal IgG against AIF (sc-5586, 1:500 dilution), rabbit polyclonal IgG against p53 (sc-6243, 1:500 dilution), goat polyclonal IgG against β-actin (sc-1616 HRP, 1:2500 dilution), in PBS containing 0.1% Tween-20. The primary antibody was removed and the membranes were extensively washed with PBS/Tween-20. The membranes were then incubated for 2 h at 4°C with 1:5,000 dilution of respective horseradish peroxidase-conjugated secondary antibodies (goat anti-mouse or anti-rabbit IgG). After removal of the secondary antibody and PBS buffer washings, the blotted membranes were incubated with ECL substrate solution (ECL™ Prime Western Blotting Detection Reagent). The densities of the specific cytochrome c, Bcl-xl, Bax, AIF, p53 and β-actin bands were visualized and captured using ImageQuant™ 400 (GE Healthcare).

### Measurements of caspase activities

#### Caspase 8 and 9

After treatment with PEITC for 3 and 6 h, the cultured cells were trypsinized, and adjusted to 10^6^ cells for each reaction. Cell pellet was lysed with cell lysis buffer on ice for 10 min, centrifuged, and then 50 μl of the supernatant was transferred to individual black microplate wells. The sample in each well was mixed with 50 μl of 2x reaction buffer (containing 10% glycerol; 0.5 mM EDTA; 20 mM HEPES, pH 7.0; DTT 10 mM) and fluorogenic Ac-LEHD-AFC, a caspase 9 substrate (50 μM) or Z-IETD-AFC (EMD Millipore), a caspase 8 substrate (50 μM). Reaction mixtures were incubated for 8 h at 37°C in dark and fluorescent signals were read using the Gemini XPS fluorescent plate reader with the excitation and emission wavelengths of 400 and 505 nm, respectively.

#### Caspase 3

Cell lysates were prepared as above and mixed with the reaction buffer (containing 0.2% CHAPS; 4 mM EDTA; 20 mM PIPES, pH 7.4; DTT 10 mM and 0.2 mM Z-DEVD-AMC), EnzCheck® Caspase-3 Assay kit#1 (Molecular Probe). Reaction mixtures were incubated for 1 h at 30°C in dark and fluorescent signals were read using the Gemini XPS fluorescent plate reader with the excitation and emission wavelengths of 342 and 441 nm, respectively.

### Statistical analysis

All the results were presented as the mean ± SEM. Statistical comparison between control and treated group was performed using Student’s t-test or One-way ANOVA with Student-Newmann-Keuls post-hoc test, where appropriate. The level of significance was set at *p* < 0.05.

## Results

### Cytotoxic effects of PEITC on CCA and Chang cells

**Figure 1:**
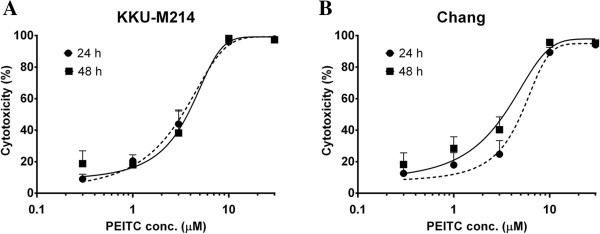
**Cytotoxic effects of PEITC on KKU-M214 and Chang cells.** Cells were incubated with various concentrations from 0.3 to 30 μM PEITC for 24 and 48 h. The cytotoxicity of PEITC in KKU-M214 **(A)** and Chang cells **(B)** was examined by SRB assay. Each value represents the mean ± SEM of three experiments.

KKU-M214 and Chang cells were exposed to PEITC at the indicated concentrations and the cytotoxicity of PEITC was assessed at 24 and 48 h. Viability of both cell lines was reduced rapidly after exposure to PEITC and the percent cytotoxicity remained similar level after 24 and 48 h incubation (Figure 1). The IC_50_ values were not different between 24 and 48 h of incubation (2.99 ± 0.87 and 3.25 ± 0.19 μM), respectively, for KKU-M214 (Figure 1A), and (3.46 ± 0.20 and 3.39 ± 0.75 μM) for Chang cells (Figure 1B). KKU-M214 was apparently more sensitive to PEITC than Chang cells, especially at 24 h of incubation.

### PEITC-induced apoptosis in relation to apoptosis-associated proteins expression

**Figure 2:**
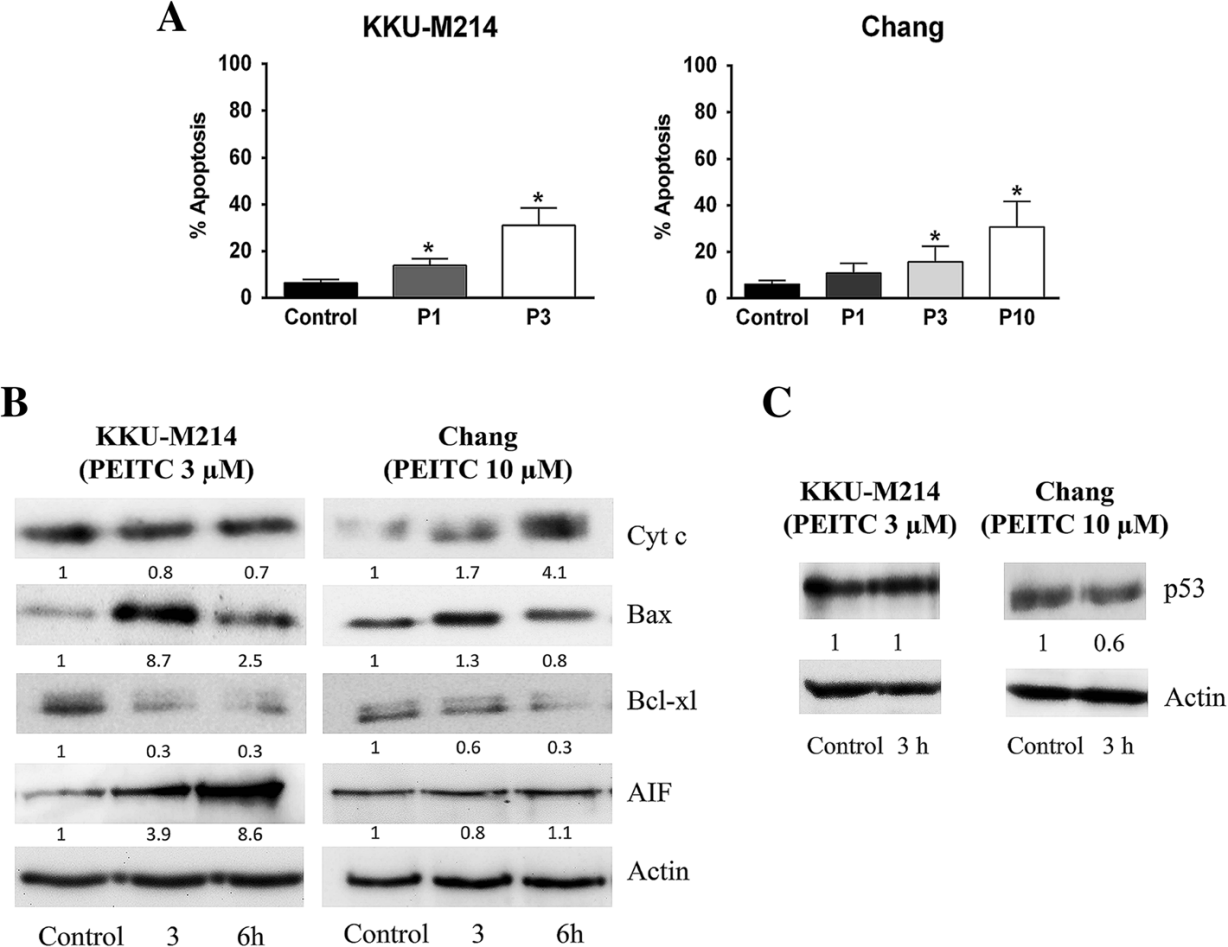
**PEITC-induced apoptosis and release of proapoptogenic proteins.** KKU-M214 and Chang cells were treated with PEITC 1, 3 or 10 μM (P1, P3 or P10, respectively) for 3 or 6 h. The number of apoptotic cells was assessed by AO/EB method at 3 h of incubation **(A)**. Each bar represents the mean ± SEM of three experiments. Significantly different from control, *p < 0.05. Immunoblot analysis was performed using 30 μg of protein and cytochrome c, Bax, Bcl-xl, AIF and p53 were detected by specific antibodies. β-actin was used as loading control **(B & C)**. Value indicates the relative protein intensity as a ratio to the corresponding vehicle-treated control. Each experiment was done at least twice with similar results, and the representative bands from one experiment are shown.

Induction of apoptotic cell death by PEITC was examined for KKU-M214 and Chang cells. PEITC induced apoptosis of both cell lines very rapidly within 3 h in a dose dependent manner (Figure 2A & B). In contrast, PEITC did not induce necrotic cell death at any time points examined (data not shown). The induction of apoptotic cell death was associated with changes in apoptotic proteins, *i.e.*, decrease of Bcl-xl and increase of Bax protein expression within 3 h. PEITC induced cytochrome c release in Chang cells but not in KKU-M214 cells. Conversely, AIF was markedly increased in KKU-M214 cells but not in Chang cells. Bcl-2 protein expression is known to be regulated partly by p53, but, in this study, no significant changes in p53 protein expression were observed at 3 h (Figure 2C) in spite of significant changes in Bcl-xl, Bax and other apoptogenic proteins in both cells.

### PEITC-induced cell death via caspase-dependent and -independent pathways

**Figure 3:**
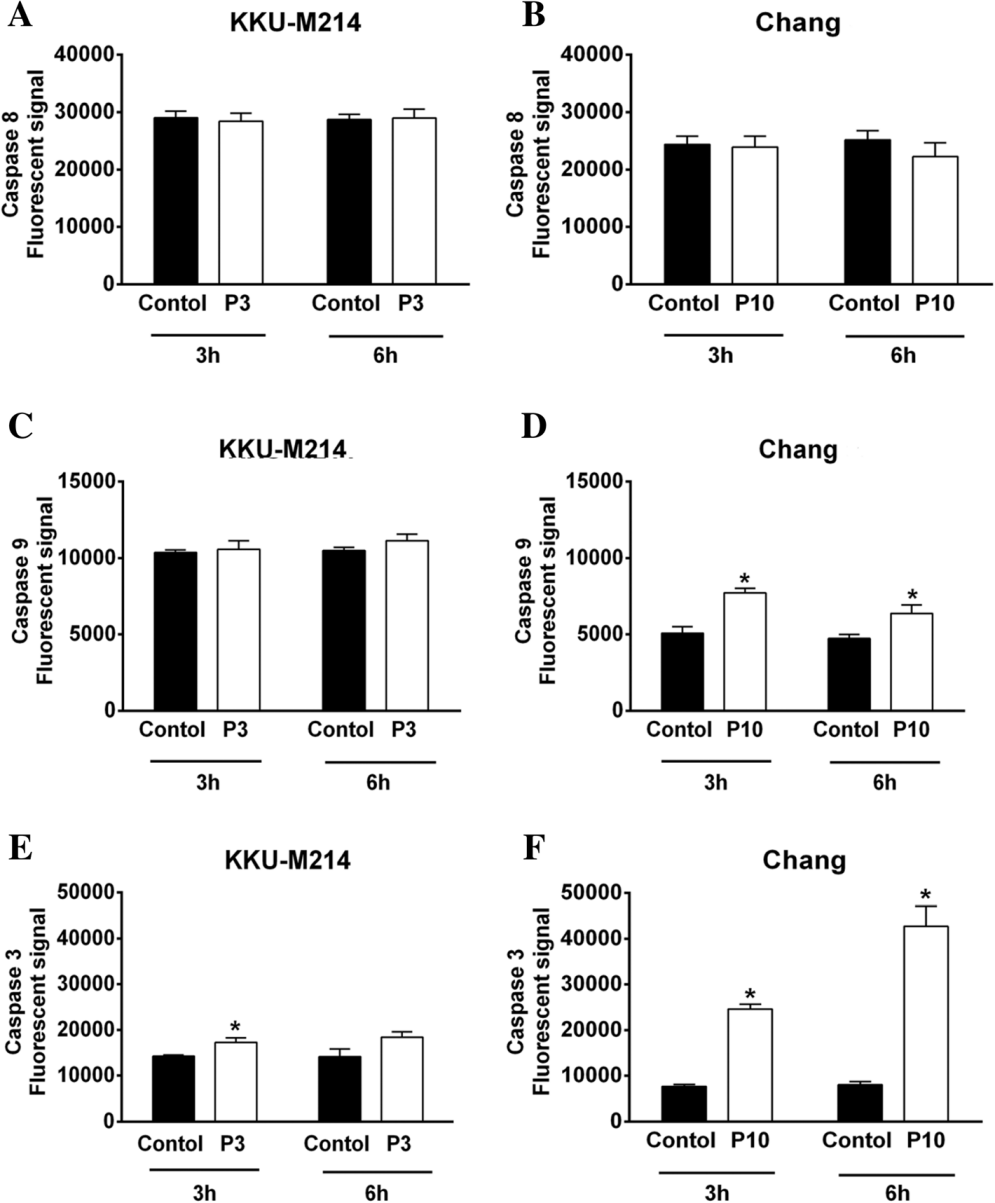
**Activation of caspases by PEITC.** Cells were treated with 3 or 10 μM of PEITC (P3, P10), for 3 and 6 h. Caspase-3 **(E & F)**, caspase-8 **(A & B)** and caspase-9 **(C & D)** activities were determined by incubating the cell lysates with fluorogenic substrates specific for each caspase enzyme. Each bar represents the mean ± SEM of three experiments. Significantly different from corresponding controls, *p < 0.05.

As the increase of AIF and cytochrome c levels are known to be involved in the intrinsic death pathway, their downstream caspase activities in mitochondrial pathway were evaluated along with caspase-8 extrinsic pathways. The effects of PEITC on caspase 3, 8 and 9 activities in both cell lines were measured at 3 and 6 h after treatment. Caspase-8 activity was unaltered in both cell lines (Figure 3A & B). While caspase-3 and -9 activities in KKU-M214 cells were unchanged after PEITC treatment, they were significantly increased in PEITC-treated Chang cells (Figure 3C-F).

### PEITC-induced glutathione depletion

**Figure 4:**
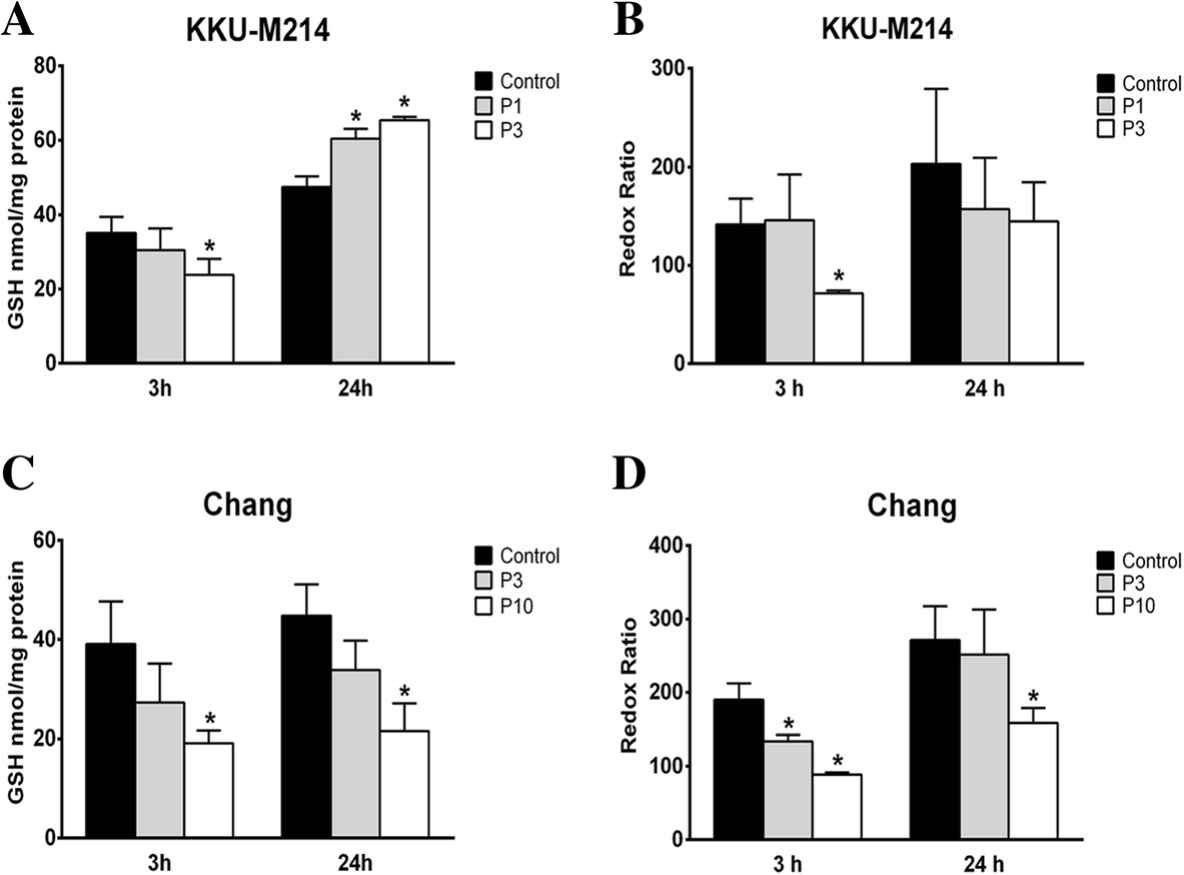
**Effect of PEITC on intracellular GSH and redox ratio.** KKU-M214 **(A & B)** and Chang cells **(C & D)** were incubated with PEITC 1, 3 or 10 μM (P1, P3 or P10, respectively) for 3 and 24 h. Total GSH contents were measured from cell pellets as intracellular GSH **(A & C)**, and GSH and GSSG ratios **(B & D)** were determined for GSH redox ratio. Each bar represents the mean ± SEM of three experiments. Significantly different from control, *p < 0.05.

Previous reports [11,12] suggested that cytotoxicity of PEITC is related to oxidative stress. As GSH is a major cellular antioxidant, we investigated the effect of PEITC on cellular GSH levels. After exposure to PEITC, both KKU-M214 and Chang cells rapidly lost cellular GSH in a dose-dependent manner as early as 3 h of incubation (Figure 4A & C). In KKU-M214 cells, GSH levels returned to, or rose up even higher than, the control level at 24 h. GSH /GSSG ratio in KKU-M214 cells was initially reduced and then returned to the control level by 24 h (Figure 4B). After treatment with 10 μM PEITC, only very few KKU-M214 cells were left alive at 24 h, then it was not possible to determine the levels of GSH. In contrast to the rapid recovery of KKU-M214 cells, PEITC-mediated depletion of GSH levels and depressed GSH redox ratios in Chang cells persisted even at 24 h of incubation (Figure 4C & D).

### Effects of antioxidants on PEITC-induced oxidative stress

**Figure 5:**
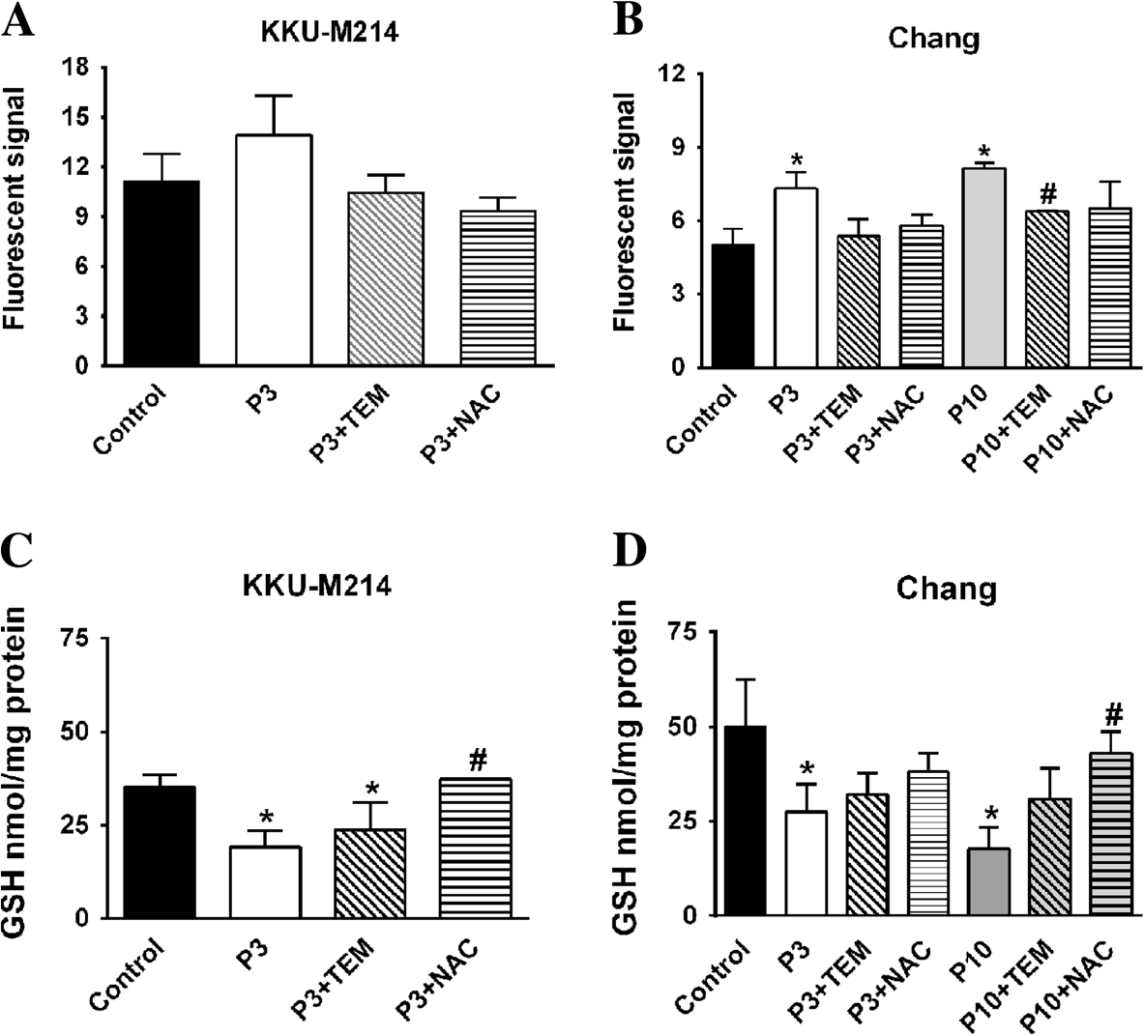
**Effect of PEITC on ROS production and GSH depletion.** KKU-M214 **(A & C)** and Chang cells **(B & D)** were incubated with PEITC at indicated concentrations 3 and 10 μM (P3 & P10) with or without TEMPOL (0.5 mM TEM) or NAC (2 mM). ROS formation was quantified by dihydroethidium method after incubation for 90 min **(A & B)** and cellular GSH **(C & D)** was assayed at 3 h after incubation. Each bar represents the mean ± SEM of three experiments. Significantly different from control, *p < 0.05, and significantly different from PEITC treated group, #*p* < 0.05.

Since the results given above, PEITC treatment induced GSH depletion in both cell lines, we examined whether this depletion was associated with the formation of reactive oxygen species (ROS). We examined also the role of antioxidants on GSH depletion and ROS formation. For this purpose, we used TEMPOL, a ROS scavenging agent, and NAC, a thiol modifier. As shown in Figure 5A and B, the basal level of superoxide in KKU-M214 cells was approximately 2-fold higher than that in Chang cells. Treatment of the cells with 3 and 10 μM PEITC caused significant increase of ROS in Chang cells, but only slight increase in KKU-M214 cells. Co-treatment of the cells with PEITC and 0.5 mM TEMPOL or 2 mM NAC completely normalized the ROS levels in both cell lines (Figure 5A & B). Moreover, treatment of NAC also largely prevented PEITC-induced losses of GSH in both cell lines at 3 h (Figure 5C & D) and this protective effect persisted up to 48 h of incubation (data not shown). However, TEMPOL, which could completely neutralize ROS, could only partially prevented GSH depletion in both cell lines.

### PEITC-induced intracellular calcium mobilization

**Figure 6:**
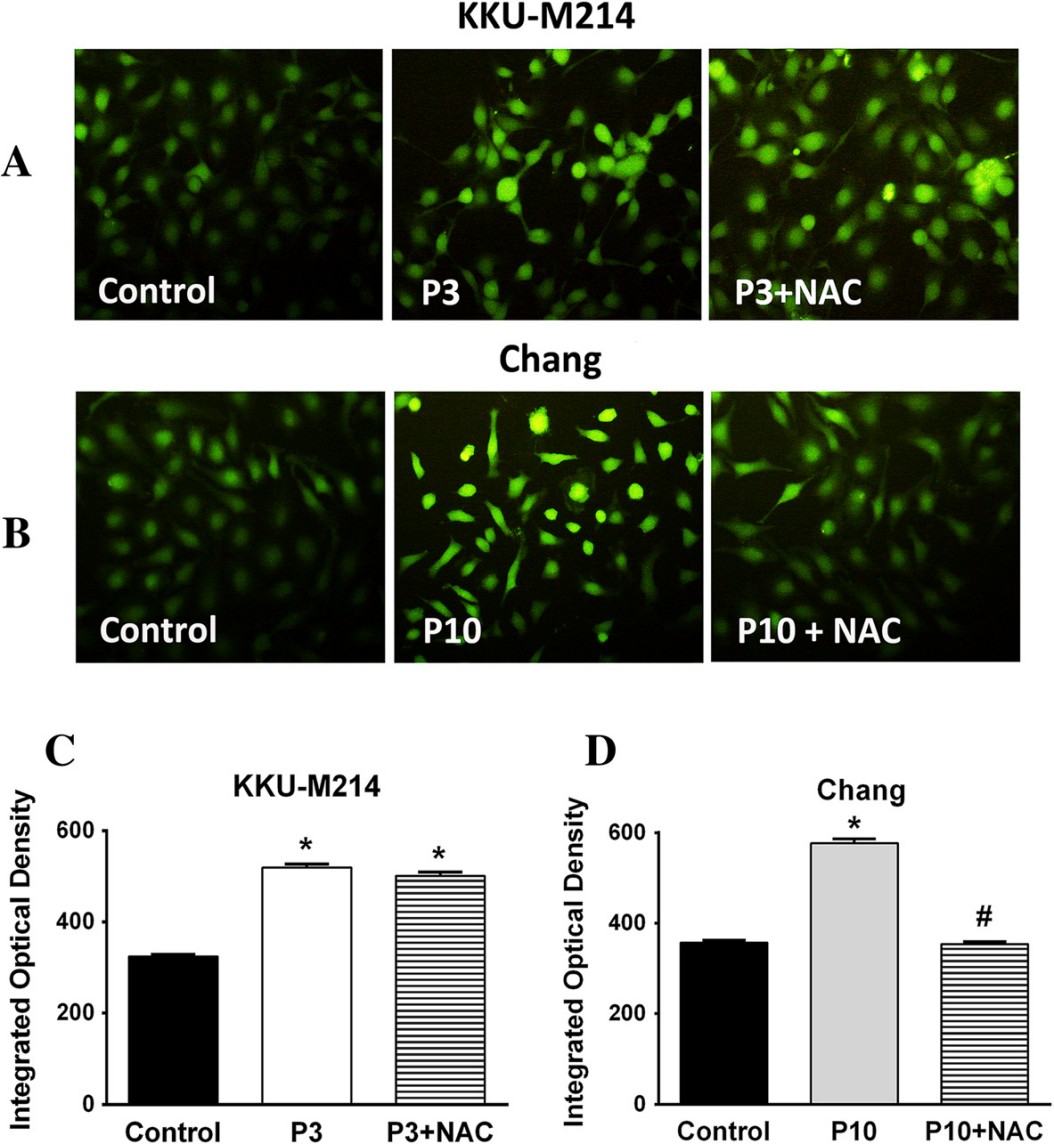
**PEITC induced the mobilization of intracellular Ca**^**2+ **^**.** Cultured cells were treated with 3 or 10 μM of PEITC (P3 or P10) with NAC (2 mM) for 1 h. The mobilization of Ca^2+^ into cytosol was analyzed by fluorescence staining using Fluoforte dye with excitation and emission wavelength of 485 and 535 nm, respectively. Representatives of images in each condition are shown **(A & B)**. The integrated optical density (IOD) of fluorescence in each condition was analyzed **(C & D)** from 10 images of two experiments. Each bar represents the mean ± SEM. Significantly different from control, *p < 0.05 and significantly different from PEITC treated group, #*p* <0.05.

Oxidative stress is known to trigger the release of Ca^2+^ from some intracellular Ca^2+^ storages, particularly from the endoplasmic reticulum, resulting in the increase of cytosolic and mitochondrial Ca^2+^, which initiates cell death [24]. We examined the effects of PEITC on intracellular Ca^2+^ mobilization in KKU-M214 and Chang cells. As shown in Figures 6A & B, PEITC induced rapid Ca^2+^ mobilization into cytosol within the first 1 hour of incubation, which was visualized by Ca^2+^ fluorescent probe in KKU-M214 and Chang cells. NAC, a thiol modifier, could not inhibit Ca^2+^ flux into cytosol in KKU-M214 cells (Figure 6C), but could completely inhibit Ca^2+^ flux into cytosol in Chang cells (Figure 6D). This underlines the causal relationship between calcium flux and oxidative stress.

### PEITC-induced depolarization of the mitochondrial transmembrane potential

**Figure 7:**
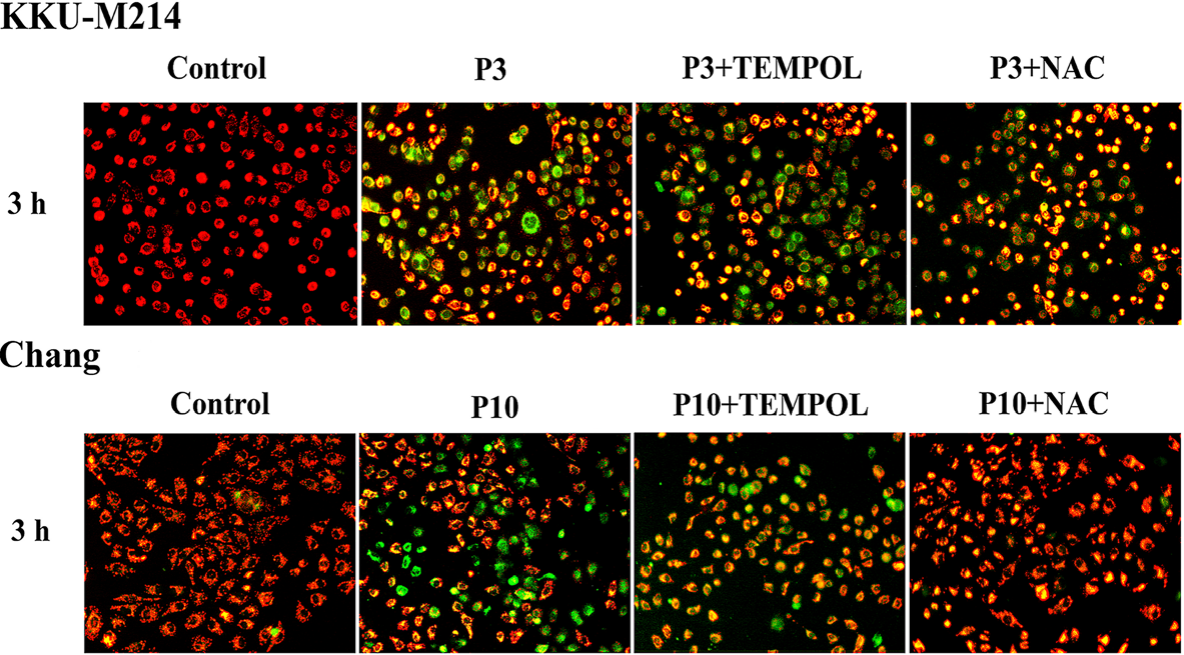
**PEITC-induced depolarization of the mitochondrial transmembrane potential.** Cells were treated with 3 or 10 μM (P3 or P10) of PEITC with TEMPOL (0.5 mM TEM) or NAC (2 mM NAC) for 3 h. The change in Δ*Ψ*_m_ was examined using JC-1 staining method. Fluorescent images were captured by fluorescence microscopy with the excitation wavelength of 485 nm and the emission wavelength of 535 nm. The experiments were performed twice with similar results, representative images from one experiment were shown.

Since PEITC induced apoptotic cell death via Bcl-2 protein family and other apoptogenic proteins, it is likely that the cytotoxicity of PEITC would be associated with the mitochondrial pathway. We examined the effect of PEITC on mitochondrial integrity by measuring the ∆*Ψ*_m_ using JC-1 fluorescent assay. In untreated control cells, mitochondria predominantly exhibited red fluorescence due to accumulation of J-aggregates representing the intact ∆*Ψ*_m_. PEITC treatment rapidly depolarized ∆*Ψ*_m_ as shown by the green fluorescence of JC-1 monomeric forms present in the cytosol (Figure 7). The effect was apparent within the first 1 hour of incubation and sustained up to 24 h in both cells. The images of the cells treated with PEITC for 3 h are shown in Figure 7. The effects of TEMPOL and NAC on the PEITC-induced ∆*Ψ*_m_ changes were evaluated. As was expected, TEMPOL did not prevent the depolarization of ∆*Ψ*_m_ in both cell lines. In contrast, NAC completely protected PEITC-induced mitochondrial depolarization in Chang cells, but this protective effect was not apparent in KKU-M214 cells, despite that GSH in KKU-M214 cells was well maintained by NAC.

### Effect of cyclosporine on PEITC-induced cell death

**Figure 8:**
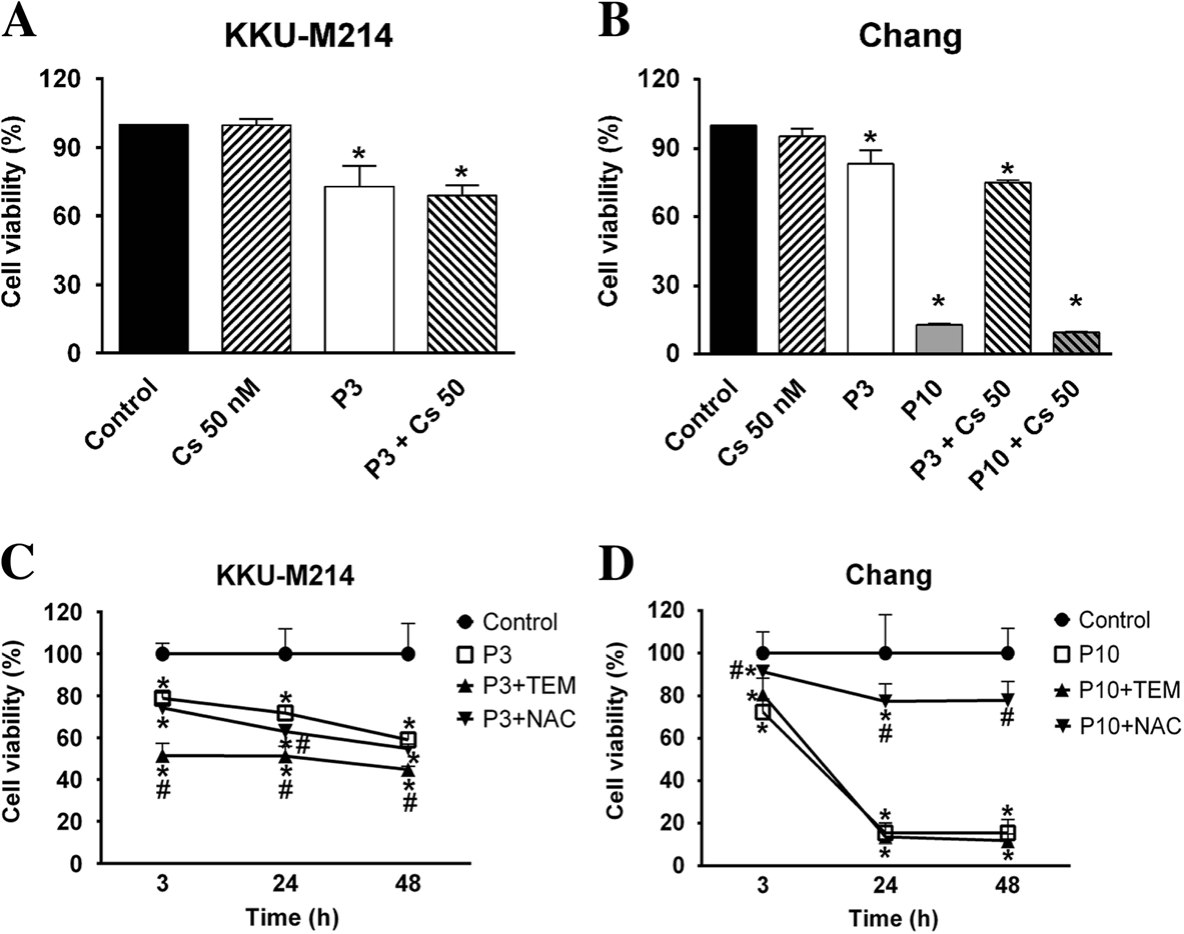
**Effects of Cs, NAC and TEMPOL on PEITC-induced cell death.** Cells were treated with 3 or 10 μM of PEITC (P3 or P10) in combination with Cs (cyclosporine) (50 nM) for 24 h **(A** and **B)**. Another experiments, cells were treated with NAC (2 mM) or TEMPOL (0.5 mM, TEM) for 3, 24 and 48 h **(C** and **D)**. Cell viability was assayed. Each bar or each value represents the mean ± SEM of three experiments. Significantly different from control group, *p < 0.05. and significantly different from PEITC treated group, #*p* <0.05.

Since the depolarization of ∆*Ψ*_m_ may be resulted from the opening of the mitochondrial permeability transition (MPT) pores, we examined whether the opening of MPT was the primary effect of PEITC to induce cell death. The results show that cyclosporine (Cs) (Sandimmune, Novartis), a potent MPT inhibitor, could prevent the losses of ∆*Ψ*_m_ (data not shown), but could not prevent cell death in both cell types (Figure 8A & B). These results suggested that the loss of ∆*Ψ*_m_ was not a critical event and might be secondary to the recruitment of Bcl-2 protein members (Figure 2).

### Effect of antioxidants on PEITC-induced cytotoxicity

It is apparent from the results given above that PEITC affected differently on KKU-M214 and Chang cells over the induction of GSH redox stress, protection of Ca^2+^ efflux into cytosol and the protection to the loss of ∆*Ψ*_m_ by NAC. Therefore, the protective effects of antioxidants, TEMPOL or NAC, on PEITC-induced cytotoxicity were assessed. Figure 8 (C & D) shows that, co-treatment of the cells with PEITC and TEMPOL apparently could not protect cell death in both cell types, which was consistent with the results of the inefficacy of TEMPOL to prevent depolarization of ∆*Ψ*_m_ changes. Rather, treatment with TEMPOL exacerbated cell death in KKU-M214 cells (Figure 8C). NAC, which was unable to prevent the loss of ∆*Ψ*_m_ nor the Ca^2+^ mobilization in cytosol in KKU-M214 cells, also could not protect PEITC-induced cell death. In contrast to the inefficacy to KKU-M214 cells, NAC almost fully protected Chang cells from the cytotoxic effect of PEITC at any time points examined after incubation (Figure 8D).

## Discussion

The chemopreventive properties of dietary cruciferous vegetables are well recognized from the results of epidemiological and experimental studies [5,6,9]. PEITC, one of the most promising ITCs, has been extensively studied *in vivo* and *in vitro*, information of its effects on CCA cells is lacking. Many strategies to enhance therapeutic outcomes in CCA treatment have been studied. For example, addition of biologic agents to block various kinase enzymes, or to suppress cytoprotective enzymes; NQO1 and HO-1 in CCA cells could increase the susceptibility of CCA to chemotherapeutic drugs [1,20,23]. In the present study, we demonstrated that PEITC could inhibit CCA cell growth and rapidly induce apoptosis. PEITC exerts different effects on KKU-M214 and Chang liver cells over cellular GSH redox and the release of mitochondrial apoptogenic molecules. The different cytoprotective effect of NAC on PEITC-induced cell death of the two cell types may reflect that the intracellular targets of PEITC are different in KKU-M214 and Chang liver cells.

Previous studies showed that PEITC induced cell death via several different mechanisms depending on cell types. Induction of cell death was associated with activation of c-Jun-N-terminal kinase (JNK) in DU145 but not in LnCaP cells [13] or with formation of ROS in PC3 and LnCaP, but was independent of ROS in HepG2 and multiple myeloma cells [12,25,26]. In this study, cytotoxic effects of PEITC were explored using a CCA cell line, KKU-M214 cells and Chang liver cells, since most chemotherapeutic agents have little selectivity over cancer cells from normal host cells. Our findings of the lack of selective toxicity of PEITC over CCA and Chang cells is consistent with the previous reports that various ITC killed cancer cells and non-cancer cells at the same order of concentration [16].

Present study showed that PEITC could induce apoptosis of both KKU-M214 and Chang liver cell lines in association with the decreased Bcl-xl and increased Bax expressions. It is known that p53 plays an important role in physically and functionally interacting with Bcl-2 family members for their translocation to mitochondria [27]. However, in the present study, the changes of the Bcl-2 protein members were not associated with p53 expression. This may imply that the apoptotic signal from PEITC to mitochondria is not transmitted via p53 pathway. Alternatively, stress signals provoked by PEITC may induce Bcl-2 family proteins via TNF family receptors, endoplasmic reticulum stress pathway or others [14,28]. It has been shown that PEITC sensitized HN22 oral carcinoma cells to DR5-mediated extrinsic death pathway [14]. We measured caspase 8 and 9 activities, which represent the initiator caspases of the extrinsic and intrinsic death signaling pathways, respectively. From the results of this study, PEITC-induced cell death appeared to be associated only with the intrinsic mitochondrial pathway, as there was no change in the caspase 8 activity after PEITC treatment.

In the present study, the cytotoxicity of PEITC was mediated via caspase-independent and caspase-dependent pathways for KKU-M214 and Chang cells, respectively. AIF is released from mitochondria and translocated to the nucleus where it fulfills the lethal function. Similar to cytochrome c, AIF play an important role in mitochondrial respiratory chain and is required for cell survival [29]. However, AIF is not a widespread cell death effector and its contribution to the execution of cell death is dependent upon the cell type, as well as the insulting signals [29]. PEITC induced AIF release in U2 OS sarcoma [11] and KKU-M214 cells in the present study. On the other hand, PEITC induced cytochrome c release in many cancer cells including MCF7, a breast cancer cell line [30], HT29, a colon cancer cell line [30], PC3, a prostate cancer cell line [12] and Chang cells in the present study. Our results showed that PEITC exerts its effects via AIF or cytochrome c depending on the cell types.

One of the prominent effects of PEITC is the induction of oxidative stress in cancer cells, which is characterized by ROS formation, GSH depletion and protein oxidation [11,12]. Our results only partially concurred with those previous reports. In the present study, PEITC induced GSH depletion in both KKU-M214 and Chang cells. However, PEITC induced ROS formation and GSH redox stress only in Chang cells but not in KKU-M214 cells (at least at 24 h of incubation). PEITC-induced cytotoxicity on Chang cells was associated with the depression of cellular GSH redox, as the replenishment of GSH by NAC could protect from cell killing by PEITC. This suggests PEITC-induced cell death of Chang cells may be via GSH redox stress. On the other hand, KKU-M214 cells seemed to handle PEITC-induced GSH redox stress rather well, because GSH levels were restored to the control level, or overshoot higher than controls at 24 h. Moreover, the treatment with NAC did not prevent PEITC-induced cell death of KKU-M214 cells. It is, therefore, the main mechanism of PEITC-induced cytotoxicity in KKU-M214 may be not by GSH redox stress. Although the cytotoxicity of PEITC against KKU-M214 and Chang cells was not much different in terms of the potency and efficacy, the underlining mechanism of the expression of cytotoxicity of PEITC on those two cells was obviously different because of the opposing results of the protective effect of NAC. It is, therefore, important to investigate further whether CCA tissues in the patients have the similar characteristics as the tumor cell responses to PEITC, before implementing NAC as an adjuvant.

PEITC is known to induce ROS formation, which is assumed to be a main mechanism of cell killing action of PEITC on some tumor cells [11,12,17]. However, in this study, ROS was not causally related to GSH depletion or induction of cell death of KKU-M214 and Chang cells. Treatment with TEMPOL, which could completely normalize the superoxide to the background level, could not prevent cell death of both types of cells. Our results were consistent with the previous report of the failure of using various radical scavengers to prevent PEITC-induced cell death [26]. These results suggest that the mechanism of PEITC to induce cell death may be unique. The possible mechanisms of PEITC could be such as, modifications of redox-sensitive proteins or forming electrophiles attracting some critical proteins [31].

Oxidative stress can trigger the mobilization of Ca^2+^ into cytosol, where endoplasmic reticulum is the important Ca^2+^ storage [24]. We observed a rapid flux of Ca^2+^ into cytosol shortly after PEITC treatment. The rapid increase of cytosolic Ca^2+^ may cause elevation of mitochondrial Ca^2+^ and decrease of Ca^2+^ in endoplasmic reticulum, and such imbalance of Ca^2+^ may trigger a variety of cascades leading to cell death. The lack of protective effect of NAC on cytosol Ca^2+^ flux in KKU-M214 cells suggests that PEITC may exert the effect on the release of Ca^2+^ by cellular stress other than the GSH redox system. On the other hand, in Chang cells, GSH redox disturbance may be primarily involved in cytosolic Ca^2+^ flux.

In the present study, an increase of cytosolic Ca^2+^ was accompanied with the rapid loss of ∆*Ψ*_m_. The depolarization of ∆*Ψ*_m_ is resulted from the opening of MPT pores, which is formed in the inner membrane of mitochondria [32]. The opening of MPT pores leads to diminish of ATP synthesis and ensuing cell death. However, present study showed that cyclosporine, a MPT pore inhibitor could not prevent PEITC-induced cell death, although it could prevent losses of the ∆*Ψ*_m_. This may imply that the depolarization of ∆*Ψ*_m_ is probably an associated event of PEITC treatment, but is not a direct effect leading to cell death. Taken all these together, mitochondria may not be a primary target of the increase of cytosolic Ca^2+^ flux for initiation of cell death. Instead, the increased cytosol Ca^2+^ may initiate the death signals in the cytosol by activation of a variety of Ca^2+^-sensitive enzymes, such as calpain, leading to the cleavage and targeting of Bax to mitochondria [24] to activate the mitochondrial cell death pathway.

Our study revealed that PEITC induces up-regulation of Bax and down regulation of Bcl-xl. The formation of Bax pore and mitochondrial outer membrane permeabilization (MOMP) upon activation may be the critical event leading to the release of proapoptogenic proteins, cytochrome c and AIF, and ensuing cell death [33]. The mechanisms of the induction of cytosol Ca^2+^ mobilization and activation of Bcl-2 proteins by PEITC remain to be elucidated. Since the precise mechanisms of PEITC-induced cell death of KKU-M214 cells remain unclear, further study is needed to explore novel mechanisms of the expression of cytotoxicity of PEITC.

## Conclusion

In conclusion, the present results show that PEITC induced apoptosis of CCA cells and Chang cells. This process may involve induction of oxidative stress and triggering of Ca^2+^ flux, which leads to mitochondrial cell death mechanisms. Effect of PEITC on redox status of GSH may be not important for cell killing for CCA cells but it is important for maintaining cell functions in Chang cells. The different effect of PEITC on different cell types was clearly shown by the cytoprotection response to antioxidant, NAC. More study is needed using several CCA cell lines over the response to PEITC. Taken together, the present results highlighted different responses of the cells to PEITC, which may facilitate the new approaches in the study of PEITC for drug development for the treatment of CCA.

## Abbreviations

CCA: Cholangiocarcinoma; Cs: Cyclosporine; GSH: Glutathione; GSSG: Glutathione disulfide; JC-1: 5,5′,6,′-tetrachloro- 1,1′,3,3′ tetraethylbenzimidazolyl carbocyanine iodide; JNK: c-Jun-N-terminal kinase; NAC: N-acetylcysteine; PEITC: Phenethyl isothiocyanate; ROS: Reactive oxygen species; SRB: Sulphorhodamine B; ∆Ψm: Mitochondrial transmembrane potential.

## Competing interests

The authors declare that they have no competing interests.

## Authors’ contributions

VK designed the study. OT performed the experiments. VK and OT analyzed the data. VK, OT, LS, AP and UK interpreted the results. OT, VK, and LS wrote the manuscript. All authors read and approved the final manuscript.

## Pre-publication history

The pre-publication history for this paper can be accessed here:

http://www.biomedcentral.com/1471-2407/13/571/prepub
